# P2RX7B is a new theranostic marker for lung adenocarcinoma patients

**DOI:** 10.7150/thno.48229

**Published:** 2020-08-29

**Authors:** Jonathan Benzaquen, Serena Janho Dit Hreich, Simon Heeke, Thierry Juhel, Salomé Lalvee, Serge Bauwens, Simona Saccani, Philippe Lenormand, Véronique Hofman, Mathilde Butori, Sylvie Leroy, Jean-Philippe Berthet, Charles-Hugo Marquette, Paul Hofman, Valérie Vouret-Craviari

**Affiliations:** 1 Université Côte d'Azur, CNRS, INSERM, IRCAN UMR 7284, 06108 Nice, France.; 2 Laboratory of Clinical and Experimental Pathology and Biobank, Pasteur Hospital, Nice, France.; 3 Hospital-Related Biobank (BB-0033-00025), Pasteur Hospital, Nice, France.; 4 FHU OncoAge, Nice, France.; 5 Centre Antoine Lacassagne, 06107 Nice, France.; 6 Department of Pulmonary Medicine and Oncology, Pasteur Hospital, Nice, France.; 7 Université Côte d'Azur, CNRS UMR 7275 - IPMC, Sophia Antipolis, France.; 8 Department of Thoracic surgery, Pasteur Hospital, Nice, France.

**Keywords:** lung cancer, P2X7R, purinergic signaling, ATP, splice variant

## Abstract

**Rationale:** The characterization of new theranostic biomarkers is crucial to improving the clinical outcome of patients with advanced lung cancer. Here, we aimed at characterizing the P2RX7 receptor, a positive modulator of the anti-tumor immune response, in patients with lung adenocarcinoma.

**Methods:** The expression of *P2RX7* and its splice variants was analyzed by RT-qPCR using areas of tumor and non-tumor lung adenocarcinoma (LUAD) tissues on both immune and non-immune cells. The biological activity of P2RX7 was studied by flow cytometry using fluorescent dyes. Bi-molecular fluorescence complementation and confocal microscopy were used to assess the oligomerization of P2RX7. Tumor immune infiltrates were characterized by immunohistochemistry.

**Results:** Fifty-three patients with LUAD were evaluated. *P2RX7A,* and 3 alternative splice variants were expressed in LUAD tissues and expression was down regulated in tumor versus adjacent non-tumor tissues. The protein retained biological activity only in immune cells. The *P2RX7B* splice variant was differentially upregulated in immune cells (*P* < 0.001) of the tumor and strong evidence of oligomerization of P2RX7A and B was observed in the HEK expression model, which correlated with a default in the activity of P2RX7. Finally, LUAD patients with a high level of *P2RX7B* had non-inflamed tumors (*P* = 0.001).

**Conclusion:** Our findings identified P2RX7B as a new theranostic tool to restore functional P2RX7 activity and open alternative therapeutic opportunities to improve LUAD patient outcome.

## Introduction

Lung cancer is the leading cause of cancer-related deaths in the world. More than two thirds of lung cancers are diagnosed at an advanced stage 1 and need medical treatment including chemotherapy, targeted therapies and immunotherapies. Despite these new approaches, the 5-year survival rate of patients with any type of lung cancer remains at around 20%, all stages combined and ranges from 57% in localized lung cancers to 5% in metastatic lung cancers 1. Therefore, deciphering new theranostic tools and understanding processes involved in the development of pro- or anti-tumor micro-environments are of crucial importance to improve patient outcome.

Purinergic receptors for extracellular nucleotides (ATP, ADP, UTP, UDP) and the nucleoside adenosine have attracted growing interest since the discovery that inflammatory and cancer tissues contain high levels of extracellular ATP (eATP) 2 and adenosine (its degradation product), which has been described as an immunosuppressor 3. The purinergic receptor family is divided into two major families, P2X and P2Y 4. Depending on the receptor involved, extracellular purines orchestrate either immunostimulation or immunosuppression of host cells, as well as proliferation or cytotoxicity of tumor cells 5. We focused on P2RX7, an ubiquitous receptor 6 described to be expressed at a particularly high level in white blood cells from the immune system, especially in monocytes/macrophages, lymphocytes and dendritic cells 7-10.

The full length P2RX7 receptor is an ATP-gated ion channel composed of three protein subunits encoded by *P2RX7A* mRNA. Activation of P2RX7 by high doses of eATP leads to Na^+^ and Ca^2+^ influx and, after prolonged activation, to the opening of a larger conductance membrane pore, also defined as macropore activity or macropore opening 11. One consequence of this macropore opening, a unique characteristic of P2RX7, is to induce cell death in eATP rich micro-environments. This feature is linked principally to the presence of a long C-terminal domain 12.

The use of transplantable murine tumor models has demonstrated that P2RX7 expressed by host immune cells coordinates anti-tumor immune responses 13,14. The expression of a functional P2RX7 receptor on human white blood cells and in human hemopathies has been largely documented in the literature 15-17. However, whereas numerous publications claimed that P2RX7 is over-expressed in solid tumors, on the basis of P2RX7 immunostaining using mostly antibodies directed against either the extracellular loop 18,19 or the C-terminal domain 20-26, only a little data are available regarding functional P2RX7 in *ex vivo* human solid cancer cells. This is of particular importance considering that P2RX7 functionality can be affected by single nucleotide polymorphisms (SNP). Over the 13252 SNPs described within *P2RX7* gene in 2020, the phenotype of 16 non-synonymous SNPs has been studied, some of which leading to numerous pathologies including infectious, bone, neuro-psychiatric, inflammatory, cardiovascular and cancerous diseases, but also poorer survival of cancer patients 27,28. In addition, we found 27 SNPs located to the boundary of mRNA splicing sites within intronic regions which may affect splicing events. However, these SNPs are too sparsely expressed to envisage that they could be involved in diseases 29.

The function of P2RX7 can also be affected by alternative splicing. Nine alternative splice variants were identified 30,31. The function of only three of them, P2RX7H with a deletion in the first transmembrane domain (ΔTM1) leading to a loss of ion channel function, P2RX7B with a deletion in the C-terminal domain (ΔCt) leading to a loss of large pore forming function, and P2RX7J with a deletion in the second transmembrane domain leading to a negative dominant isoform, have been studied in more detail 18,19,30-33.

P2RX7 protein expression in lung adenocarcinoma (LUAD) was reported by Boldrini et al. 34, but it is currently unknown whether functional P2RX7 is expressed in LUAD. In this study we aimed at deciphering the expression of P2RX7 and its splice variants, and the implication of such variants on the biological function of the receptor and on inflammatory infiltration of tumors.

## Materials and Methods

### Patients

Twenty-four bronchopulmonary samples were obtained from the middle of the tumor and from non-tumor tissue for each patient operated on for early stage (I-IIIA) LUAD. The diagnosis and the margin of the samples were confirmed as tumoral by two lung cancer pathologists. The median age was 68 years-old and 79% of the patients were smokers or former smokers. In the retrospective study, twenty-nine LUAD samples were obtained from the biobank of the Nice University Hospital (Clinical and Experimental Pathology Laboratory) and were taken from patients operated on at the thoracic surgery department of the Nice University Hospital for early stage (I-IIIA) LUAD. The median age was 67 years-old and 100% of the patients were smokers or former smokers (see **Table 1**). Free and informed consent was obtained for each patient with the agreement of the South East CPP.

### Tumor processing

After surgery, tumor tissues were kept in RPMI (Roswell Park Memorial Institute medium, Gibco®) at 4°C, for a maximum of 12 h. Sample preparation included scissor dissociation, incubation in Human Tumor Dissociation Kit (MACS®) buffer at 37°C for 40 min and washed by DPBS 10% FBS. Cells were counted and a trypan blue test was performed to ensure cell viability. Cells were magnetically sorted (mouse anti-CD45 MicroBeads, Miltenyi®) according to the manufacturer's procedure. A sorting enrichment control experiment was performed using a humanized anti-CD45 AF488 coupled antibody (Miltenyi®) after Fc blocking (**Figure S1A**).

### Immunofluorescence

After tumor processing as described in the dedicated section, the cell suspension was homogenized, 500 000 LUAD cells were plated on slide and fixed by drying at room temperature (RT) and stained. To ensure that cells came exclusively from the intratumor area of LUAD, the margins were checked on the corresponding tissue blocks and confirmed as adenocarcinomatous by two lung cancer pathologists. HEK293T cells were plated at 600 000 cells in a 6-wells culture plate containing coverslip poly-D-lysin (Gibco) coated and transfected as described below. Anti CD16/CD32 antibodies (Fc Block, BD Biosciences) were used (1 h, RT) to reduce the non-specific signal, and primary antibodies were added for 2 h at 4°C. After washing, secondary antibodies were added for an additional 1 h at RT. Anti-P2RX7 hybridoma (dil1/2) was used. This antibody is routinely used to detect the expression of functional P2RX7 6. Anti-CD45-FITC (dil1/50, recombinant human IgG1, REA747 clone, MACS®) and anti-goat Alexa fluor 594, (1/200, Thermofisher) antibodies were used. The nuclei were counterstained with 4'6-Diamidine-2-phenylindole dihydrochloride (DAPI) in the mounting medium (Invitrogen Life Technologies^TM^).

### P2RX7 functional assay

The overall cellular P2RX7 activity was evaluated by flow cytometry. This assay allows simultaneous study of live cells stimulated with BzATP (a stable analogue of ATP), for variations in calcium levels (FLUO-4-AM dye) and macropore opening (TO-PRO-3 dye) (**Figure S3**). In practice, cells were incubated in functional assay buffer (sucrose 300 mM, KCl 5 mM, MgCl2 1 mM CaCl2 1 mM, glucose 10 mM, HEPES pH 7.4 20 mM) and loaded with Fluo-4-AM (500 nM), for 30 min at RT. Propidium iodide was added for the last 5 min to stain both dying cells and P2RX7's independent permeabilization events, which will be excluded from the final analysis. FLUO-4-AM and propidium iodide were removed by 2 washes with DPBS containing FBS 5%, and 400 000 cells were incubated in the functional buffer and stimulated with BzATP in the presence of TO-PRO-3 (633 nM, Invitrogen) for the indicated time. The reaction was stopped with 2 mM MgCl_2_ and washed with DPBS containing FBS 5%. Flow cytometry analysis was performed on a propidium negative population using a FACS Canto® II (BD Bioscience®) and data were analyzed using DIVA® or FlowJo® software. The normalization of fluorescence intensity was performed by retrieving at every time-point the fluorescence intensity (I) (FLUO-4-AM and TO-PRO-3) of the first time point (I_0_). Normalized data were presented as violin plot.

### RT-qPCR

Total RNA extraction from purified cell subpopulations or frozen tissues was performed using the AllPrep RNA Mini Qiagen® kit and reverse transcription of 37.2 ng total RNA was performed with the Applied Biosystems® kit. The cDNA was subjected to quantitative PCR using the Fast Sybr®Green Master Mix kit (Applied biosystems) and a StepOnePlusTM thermocycler. Relative expression was determined using the log2 fold change (2^-ΔΔCt^) method with *RPLP0* as the housekeeping gene. The oligonucleotides, designed to overlap splicing sites, are presented in **Figure S6A.** QPCR amplicons were sequenced to confirm their specificity (data not shown) and used as matrices of standard amplification curves to validate the absolute quantification rate and the amplification efficiency of each oligonucleotides couple (**Figure S6B**).

### Cell cultures and transfection

HEK293T cell line, provided by ATCC, were cultured in DMEM medium supplemented with 10% FBS and 100 U/mL penicillin and 100 mg/mL streptomycin at 37°C in a humid atmosphere containing 5% CO_2_. P2RX7A and P2RX7B splice variants were synthesized by Eurofins® and sequenced. HEK293T cells were transfected with pcDNA expression vector coding for the indicated cDNA using Lipofectamine® 3000 (Thermofisher) according to the manufacturer's instructions. “PcDNA3.1 venus 1” and “pcDNA3.1 venus 2” were provided by Dr Saccani's team. Transient expression analyses were processed 48-h post transfection. For the establishment of stable cell lines expressing the untagged P2RX7A, P2RX7B or P2RX7AB, blasticidin 1 µg/mL and puromycin 1 µg/mL were added to the growth media. After 3 weeks, clones were selected and tested for protein expression by Western blotting.

### Western blotting

Western blot analysis was performed following the standard protocol. In brief, cells were lysed on-ice in TR3 buffer (SDS 3%, glycerol 10%, Na_2_PO_4_ 10 mM + Complete™ Protease Inhibitor Cocktail, Roche), 20 to 50 µg of total proteins were subjected to 9% SDS-PAGE and transferred onto PVDF membranes (Immobilon Millipore®). Blots were than blocked for 20 minutes with 5% non-fat dried milk and incubated with primary antibodies (Rabbit anti-Human extracellular loop of P2RX7, APR-008, Alomone), overnight at 4°C, followed by three 5-min washes with Tris-Buffered Saline 0.1% Tween® 20 detergent, then incubated with species specific secondary IgG antibodies coupled to Horseradish peroxidase (HRP) (Goat anti-Rabbit, AP307P, Sigma, and Goat anti-Mouse, AP308P, Sigma) 1 h at RT, followed by 3 more washes as described before. The signal was revealed using PXi imaging (Syngene). In a second step, αTubulin (Mouse anti-tubulin, Sigma) level was monitored as a control for protein loading.

### Immunohistochemistry

Immunohistochemical (IHC) staining for CD45, CD3, CD8, CD20 and CD33, was performed on tumor sections obtained from LUAD patients of the prospective cohort. In brief, formalin fixed paraffin embedded 4 µm thick tissue sections were stained with specific anti-human antibodies on an automated staining platform (Benchmark ULTRA; Ventana) using the appropriate concentration. An OptiView DAB IHC Detection Kit (Ventana) and an OptiView Amplification Kit (Ventana) were used according to the manufacturer's recommendations for the visualization of the bound antibodies; sections were counter-stained with hematoxylin. Blinded quantification of the brown staining was done as follows: 5 different zones of the scanned tumor section (NanoZoomer 2.0 HT from Hamamatsu) were selected at low magnification (1×) and images of the selected fields were analyzed at 40× magnification. Scoring of the staining was as follows: 1 = 0-10% positive cells, 2 = 10-50% positive cells and 3 = ≥ 50% positive cells.

### Bioinformatic and statistical analysis

Quantitative data were described and presented graphically as medians and inter quartiles or means and standard deviations. The distribution normality was tested with the Shapiro's test and homoscedasticity with Bartlett's test. For two categories, statistical comparisons were performed using the Student's *t*-test or the Mann-Whitney test. Overall survival was defined as the interval between the date of diagnosis and the date of death from any cause. These data were estimated and presented graphically using the Kaplan-Meier method. The survival curves were compared using the log-rank test. All statistical analyses were performed using GraphPad Prism® v.8.0.2. Tests of significance were two-tailed and considered significant with an alpha level of *P* < 0.05. SnapGene 4.3.8 was used for plasmid construction. The results shown in **Figure 4B** and **Supplementary Figure 7** are based on data generated by the TCGA project for which we are grateful.

## Results

### P2RX7 is expressed in LUAD

**Figure 1:**
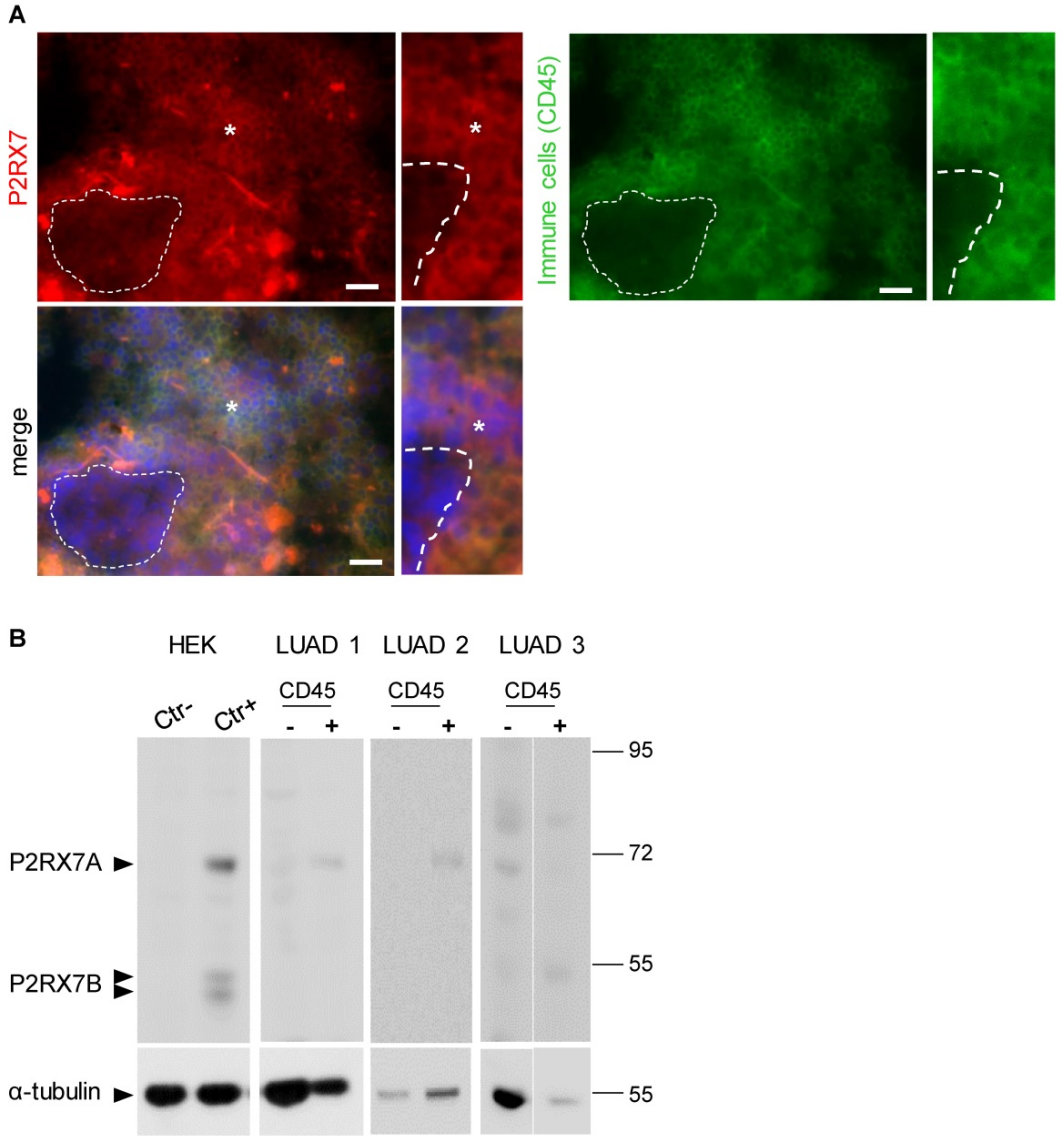
LUAD patients express P2RX7. **A.** Representative images of P2RX7 staining in LUAD tissue using the conformational anti-P2RX7 antibody, in red. CD45 staining highlighted immune cells, in green. * showed P2RX7 expressing cells. The hatched line shows CD45^-^ cells. DAPI staining shows nuclei. Magnification 20X, scale bar = 200 µm. **B.** Representative Western blot showing P2RX7 expression in HEK cells transfected with empty vector (Ctr^-^), cells transfected with both P2RX7A and P2RX7B expressing vectors and purified CD45^+^ and CD45^-^ cells isolated from tumor areas of LUAD patients using the anti P2RX7extracellular loop antibody (Alomone, APR-008, 1/1000). The total αTubulin level was monitored as a control for protein loading.

To analyze the expression levels of conformational P2RX7 in LUAD, tumor tissue was dissociated, and the cells stained with the anti-P2RX7 antibody directed against the external domain of the tertiary structure of P2RX7, which has been described to antagonize its activity 6. Whereas most of the cells were labeled, a small fraction of cells with large nuclei, a characteristic of tumor cells, were negative (**Figure 1A**). Next, an anti-CD45 antibody was used to characterize the nature of P2RX7 expressing cells and we observed that only CD45^+^ cells were stained with the anti-P2RX7 antibody. Since cells were permeabilized, the lack of staining in CD45^-^ cells was not due to intracellular retention of P2RX7 (**Figure S2**). To extend this observation, immune (CD45^+^) and non-immune (CD45^-^) cells were isolated from three LUAD specimens and subjected to western blot analysis using a second antibody which recognizes the extracellular domain of P2RX7A and P2RX7B monomers (**Figure 1B**). A protein of 70kDa corresponding to P2RX7A was found to be expressed by CD45^+^ immune cells in 2 of 3 LUAD patients. These patients only showed very limited (if any) expression of P2RX7 on tumor cells. By contrast, we observed P2RX7A expression on tumor cells of the third LUAD patient. We also noticed the presence of additional bands (from 80 to 56kDa). These bands are of undetermined nature, being not recognized by the antibody in the HEK cell expression system transfected with P2RX7A. In this patient, expression of P2RX7A was poorly detected on immune cells. Instead, a protein of 55kDa, which could correspond to P2RX7B, was observed.

Therefore, using two independent antibodies, our results showed that P2RX7A is preferentially expressed on immune cells of LUAD patients.

### The macropore function of P2RX7 is impaired in immune cells of LUAD

**Figure 2:**
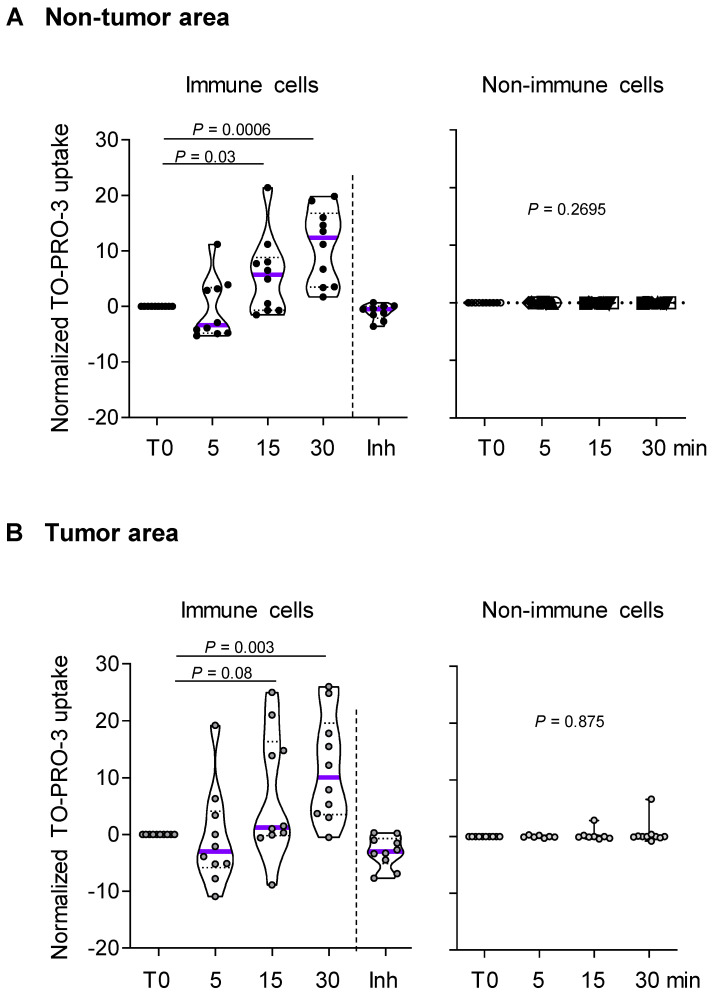
P2RX7 expressed by immune cells of LUAD patients is functional. **A.** Time course of macropore opening in purified immune and non-immune cells isolated from an adjacent non-tumor area of LUAD patients. When indicated we used a specific P2RX7 inhibitor (Inh) demonstrating that TO-PRO-3 uptake depends on P2RX7 activity. **B.** Time course of macropore opening in purified immune and non-immune cells isolated from the tumor area of LUAD tissue. The percentage of TO-PRO-3 positive cells in response to BzATP (250 µM) is shown (paired Student's *t* test). n=10 paired LUAD specimens and corresponding adjacent non-cancerous tissues from our prospective cohort. The median value is highlighted by the violet line.

Since P2RX7 expression could not predict P2RX7 activity, we investigated the macropore function of P2RX7 in both immune and non-immune cells of LUAD specimens and paired corresponding adjacent non-cancerous tissues (**Figure 2 and Figure S4**). BzATP increased the uptake of TO-PRO-3 in immune cells from both non-tumor and tumor areas (**Figure 2A-B).** This increase was prevented in cells pre-treated with the specific P2RX7 inhibitor, GSK1370319A 35, indicating that TO-PRO-3 uptake was dependent on the expression of P2RX7. In addition, the macropore activity was delayed in the tumor compartment. In fact, 15 min post stimulation, the normalized median percentage of TO-PRO-3^+^ cells was 1.2% in the tumor infiltrate (**Figure 2B**), while it reached 5.7% in the non-tumor infiltrate (**Figure 2A**). This difference was still observed 30 min post stimulation. These results suggest that P2RX7 expressed by immune cells within the tumor area was less functional.

Considering the differential activation status of P2RX7 in immune cells compared to non-immune cells, we hypothesized that alternative splicing of *P2RX7* mRNA may generate hetero-P2RX7 trimers with altered receptor functions.

### *P2RX7B* is differentially upregulated in the LUAD immune infiltrate

**Figure 3:**
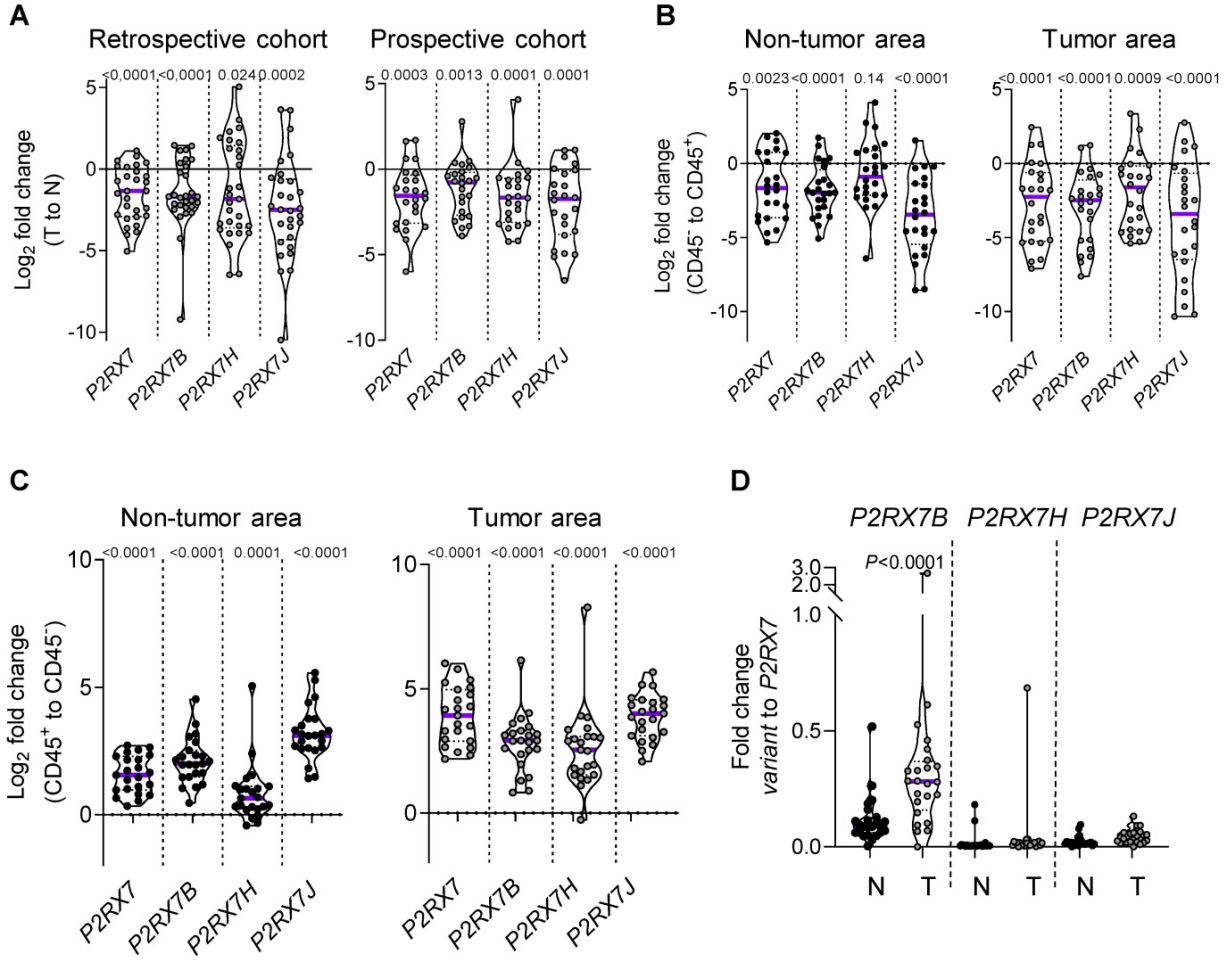
LUAD patients express *P2RX7A, B, H and J* mRNAs. **A*.**** P2RX7* splice variant expression in the retrospective (n=29) and prospective cohorts (n=24) of tumor area of LUAD tissues (T) versus adjacent non-tumor lung tissues (N). **B.** *P2RX7* splice variant expression in CD45^-^ cells isolated from adjacent non-tumor and tumor areas of LUAD patients (n=24). **C.** *P2RX7* splice variant expression in CD45^+^ cells isolated from adjacent non-tumor and tumor areas of LUAD patients (n=24). Results are expressed in Log_2_ fold change (meaning Log_2_ of 1 = 2-fold and Log2 of -1 = 0.5-fold). **D**. Differential up-regulation of *P2RX7B* in immune cells. n=24 (Mann-Whitney test).

**Figure 4:**
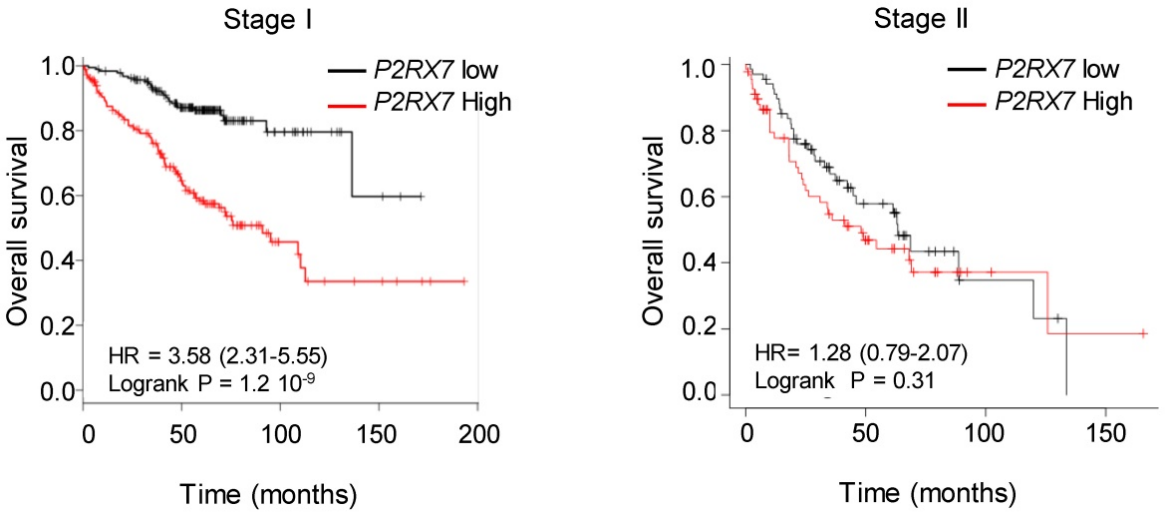
High expression of *P2RX7* is an indicator of poor survival in LUAD patients. Kaplan-Meier survival analysis (http://kmplot.com) 41 according to the *P2RX7* expression in patients with LUAD of indicated stage. High expression corresponds to value > *P2RX7* median expression, low expression corresponds to value < *P2RX7* median expression. Probability of survival of patients: Stage I: n=370 patients, low expression (n=187) median OS: 136 months; high expression (n=183) median OS: 38 months, HR: 3.58, 95% CI: 2.31-5.55, *p*=1.2 10^-9^. Stage II: n=136 patients, low expression (n=68) median OS: 63 months; high expression (n=68) median OS=38 months, HR: 1.28, 95% CI: 0.79-2.07, *p*=0.31 (log-rank test). Vertical tick-marks represent censored data.

We designed probes for the five variants described to be transcribed and translated, namely *P2RX7A, B, D, H* and *J* and analyzed their expression on human peripheral blood mononuclear cells by qualitative PCR analysis (**Figure S5A**). PBMCs expressed the *P2RX7A, B, H* and *J* variants (**Figure S5B**). Next, using qPCR probes (**Figure S6**), we quantified *P2RX7* expression on LUAD tissues using our two in-house cohorts (see **Table 1**). Using the probe which amplifies *P2RX7A, B* and *H* (further designed as *P2RX7*), we observed that *P2RX7* was 2.5-fold less expressed in tumor versus non-tumor areas of LUAD (**Figure 3A**). Likewise, the specific expression of the *P2RX7B, H* and *J* splice variants was decreased 3.2-, 3.6- and 5.6-fold, respectively. These results were confirmed with TCGA RNA-seq expression data from a larger cohort of 57 LUAD (Figure S7). Further, we analyzed the expression levels of all P2RX7 mRNA in both non-tumor and tumor areas and observed that while *P2RX7* was down-regulated in non-immune cells (**Figure 3B**), it was up-regulated in immune cells (**Figure 3C**). In the tumor area, *P2RX7* was 15-fold up-regulated in immune cells compared to non-immune cells and the alternative splice variants *P2RX7B, H* and *J* were 7-, 6- and 15-fold up-regulated, respectively. Next, we compared the expression levels of each *P2RX7* splice variant in immune cells purified from normal and tumor tissues and showed that the *P2RX7B* variants were differentially up-regulated in the immune infiltrates of LUAD patients (37% of the constitutive splicing), whereas the other variants were almost absent (**Figure 3D**). To evaluate whether up-regulation of *P2RX7* may impact the fate of LUAD patients, we analyzed overall survival (OS) of the LUAD TCGA cohort, separated into two groups, stage I and stage II. A Kaplan-Meier analysis of patients with LUAD and a high expression of *P2RX7* had a significantly poorer OS (38 months) than those with a low expression of P2RX7 (136 months). This tendency was present only in early-stage I LUAD and disappeared at stage II (**Figure 4**). At this point, no correlation with *P2RX7B* expression levels could be done, since neither the nature of *P2RX7* splice variants nor the nature of P2RX7 expressing cells were present in TCGA database.

Collectively, these results suggest that the more *P2RX7B* was expressed, the more the macropore function was dysfunctional in the immune infiltrate of LUAD.

### P2RX7B expression negatively impacts the P2RX7 activity

**Figure 5:**
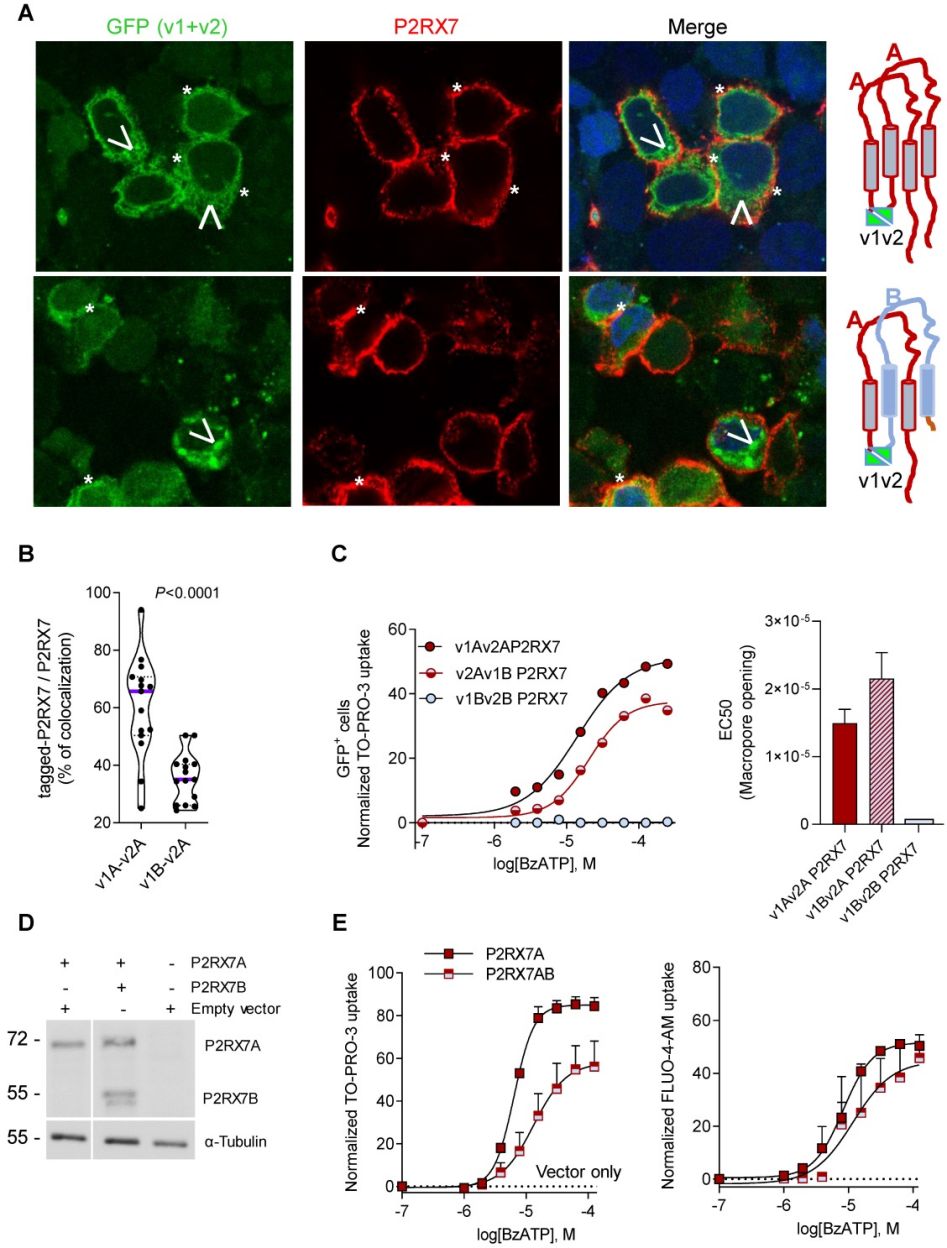
** Expression of P2RX7B decreases the overall activity of P2RX7. A.** Representative images of P2RX7 immunofluorescence in HEK cells transiently transfected with P2RX7A or with P2RX7 A + B pDNA. The tagged receptor (P2RX7A or P2RX7AB) is shown in green. The conformational receptor (P2RX7A) is shown in red. *: P2RX7 expressed at the membrane. Arrowhead: intracellular chimeric receptor. **B.** Quantification of data presented in A. n = 30 cells from 2 independent experiments. (unpaired Student's *t* test). **C.** Impact of P2RX7B expression on the P2RX7 macropore activity. HEK cells were transiently transfected with tagged-A, -B and -A+-B P2RX7 isoforms and the macropore activity was studied on GFP^+^ cells (right panel). The left panel shows the EC_50_ for macropore formation for 3 independent experiments. **D.** The expression of P2RX7A and P2RX7B was detected in stably transfected HEK clones by Western blotting using the P2RX7 anti-extracellular loop antibody. The total αTubulin level was monitored as a control for protein loading. **E.** P2RX7 activity in stably transfected HEK clones with untagged-A and -A+-B P2RX7 isoforms or vector alone (hatched line). Dose response of BzATP-induced macropore opening (left panel) and an intracellular Ca^2+^ variation (right panel).

It was previously reported that P2RX7B could heteromerized with P2RX7A 31. Therefore, P2RX7B may be co-expressed with P2RX7A and form a chimeric receptor in the immune infiltrate of LUAD. To test this hypothesis and characterize the cellular localization of such a chimeric protein, we explored the expression and the function of P2RX7AB using a bi-molecular fluorescent complementation approach. First, we controlled that cells transfected with individual hemi venus 1 (v1) or hemi venus 2 (v2) tagged-P2RX7 (P2RX7A and P2RX7B) did not emit a fluorescent GFP signal (**Figure S8A**). Thanks to the conformational anti-P2RX7 antibody, we demonstrated that individual v1- or v2-tagged P2RX7A formed homo trimers that are correctly localized at the cell membrane (**Figure S8A**). Next, we transiently co-transfected HEK cells with v1-P2RX7A and v2-P2RX7A. The interaction of the proteins brought the two hemi GFP within proximity, allowing GFP to reform its native structure and emit a fluorescence signal (**Figure 5A**). Confocal microscopy showed that v1+v2-P2RX7 was expressed at the cell surface. Furthermore, the tagged-P2RX7 adopted a normal conformation when stained with the conformational anti-P2RX7 antibody. Importantly, we verified that this antibody did not stain the P2RX7B protein (**Figure S9A**). We also noticed that some GFP^+^ small dots remained within the cytoplasm. To study the impact of P2RX7B expression on P2RX7 function, HEK cells were transiently co-transfected with v1-P2RX7B and v2-P2RX7A (**Figure 5A, lower panel**). When expressed, the chimeric GFP^+^ P2RX7AB receptor was found to be correctly inserted into the cell membrane (asterisk). The GFP chimeric proteins co-localized with P2RX7 stained with the conformational antibody (asterisk), suggesting that the chimeric receptor, like P2RX7, was inserted into the cell membrane. To confirm this finding, we quantified the percentage of tagged receptors that co-localized with P2RX7 at the membrane and observed that 35% of the chimeric protein co-localized with proteins stained with the conformational antibody (**Figure 5B and S8B**). We also observed the presence of numerous GFP^+^ aggregates within the cytoplasm (arrowhead). We then tested whether the expression of P2RX7B modified the overall activity of P2RX7. To do so, transfected cells were stimulated with increasing doses of BzATP for 15 min, GFP^+^ cells were sorted by FACS and the percentage of TO-PRO-3^+^ cells within this fraction was analyzed (**Figure 5C**). BzATP dose dependency for TO-PRO-3 uptake was shifted to the right in HEK v2Av1B P2RX7 (P2RX7AB) cells compared to HEK v1Av2A P2RX7 (P2RX7A). We then calculated the EC_50_ for macropore formation in both chimeric tagged P2RX7AB or tagged P2RX7A and P2RX7B and observed an increase of 30% (20µM vs 14 µM) in cells co-transfected with both P2RX7A and P2RX7B (F**igure 5C**). By contrast, cells transfected with v1Bv2B P2RX7 (P2RX7B) did not show any TO-PRO-3 uptake, indicating that the homo trimer P2RX7B was unable to form a macropore, despite its expression at the cell membrane and its ability to induce Ca^2+^ influx (F**igure S9B**). To go further and given the impact of the tag on membrane localization, we decided to co-express non tagged P2RX7A and P2RX7B and reevaluate the overall activity of P2RX7. To allow a meaningful comparison, we selected clones expressing comparable protein levels of P2RX7A and P2RX7B, using an antibody specific for the extracellular loop and known to recognize both P2RX7A and P2RX7B (**Figure 5D**). We then assayed both the macropore and the Ca^2+^ channel activities. Whereas the overall macropore activity of the cells transfected with both P2RX7A and P2RX7B proteins was inhibited by 50% compared to control cells transfected with P2RX7A only (EC50 =6.3 µM vs 13.1 µM and maximum TO-PRO-3^+^ cells = 58% vs 84%), we did not observe any significant effect on the intracellular Ca^2+^ concentration (**Figure 5E**). These results supported the notion that expression of the P2RX7B negatively impacted the overall macropore activity of P2RX7. At this point, we were unable to analyze the macropore activity of chimeric P2RX7AB itself. Indeed, we cannot exclude the hypothesis that the fraction of GFP^+^ cells used to assay the macropore activity also expressed functional P2RX7A homo trimers allowing the influx of TO-PRO-3 in response to BzATP. However, based on the presence of large cytoplasmic aggregates in cells expressing both P2RX7A and P2RX7B, we can reasonably propose that P2RX7A is retained intracellularly. Such cell retention could impact the quantity of functional P2RX7 expressed at the cell membrane and consequently the macropore activity. In contrast, the absence of the effect on BzATP-induced increased Ca^2+^ concentration might not be surprising considering that Ca^2+^ is able to permeate more easily than a large dye, such as TO-PRO-3, across the pore formed by P2RX7.

### Expression of *P2RX7B* correlates with decreased T cell infiltration in LUAD

**Figure 6:**
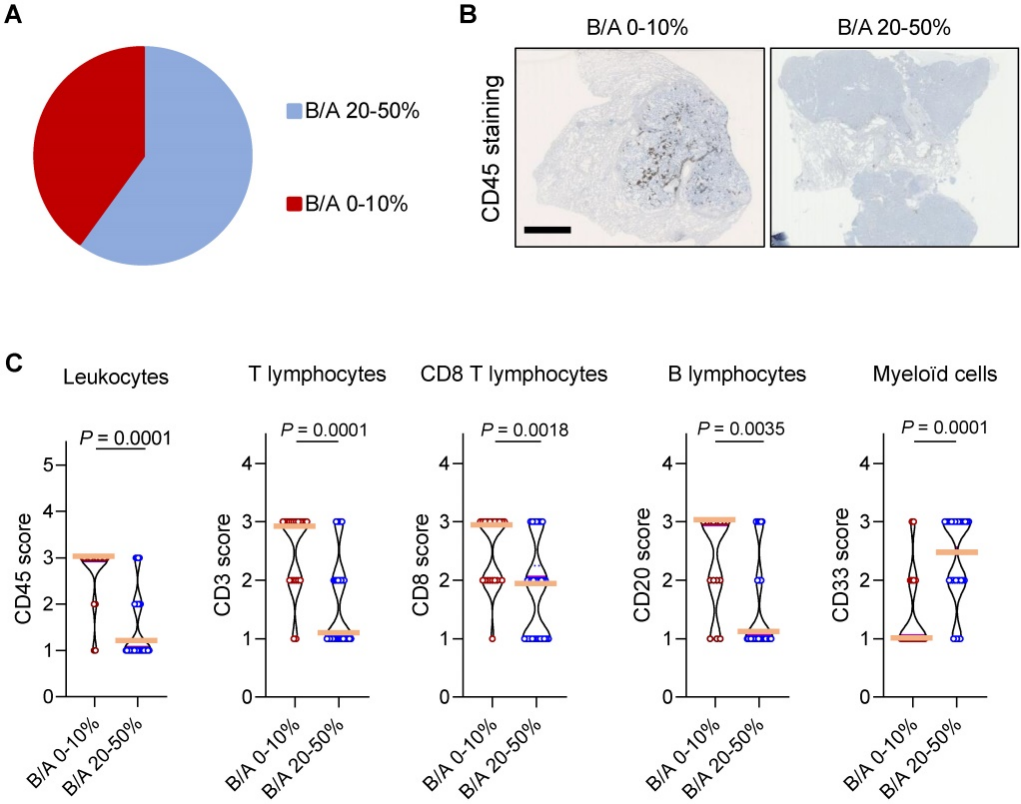
**High expression of *P2RX7B* mRNA correlates with low leukocyte infiltration in LUAD patients. A.** Clustering of LUAD patients expressing *P2RX7B* for the prospective cohort**. B.** Representative images of CD45^+^ IHC staining of samples from LUAD patients depending on the *P2RX7B* expression. Bar = 5mm. **C.** Quantification of CD45, CD3, CD8, CD20 and CD33 staining. B/A 20-50%, n=6; B/A 0-10%, n=4. (CD45, CD3 and CD8: Mann-Whitney test. CD20 and CD33: unpaired Student's *t* test).

We next wondered whether *P2RX7B* expression could impact the composition of the immune infiltrate in LUAD. Analysis of the ratio of *P2RX7B* versus *P2RX7A* mRNA expression allowed us to separate two populations within the prospective cohort (**Figure 6A**). We clustered patients expressing 1 *P2RX7B* for 2 to 4 *P2RX7A* (ratio comprised between 0.2 to 0.4), which corresponded to 60% of the specimens, from patients expressing 1 *P2RX7B* for 10 or more *P2RX7A* (ratio comprised between 0.1 to 0.0003). We then performed immunohistochemistry to qualify tumor infiltrating immune cells, focusing on leukocytes (CD45^+^ cells) (**Figure 6B**), and T lymphocytes (CD3^+^), CD8^+^ T cells, B lymphocytes (CD20^+^) and myeloid cells (CD33^+^). Quantification of the staining of these cells showed that the less *P2RX7B* is expressed the higher the number of leukocytes recruited into LUAD (**Figure 6C**). This was observed for the lymphoid lineage (T lymphocytes, CD8 T lymphocytes and B lymphocytes). However, we noticed an inverse correlation regarding the myeloid lineage (monocytes/macrophages, MDSC), characterized by cells expressing the transmembrane receptor CD33.

## Discussion

In this study we characterized the expression levels and the functionality of P2RX7 in LUAD patients and showed that whereas P2RX7 was found to be expressed in both tumor and immune cells, only immune cells expressed a functional receptor. Mechanistically, we observed differential expression of the *P2RX7B* splice variant in immune cells within tumor areas. Upregulation of P2RX7B was previously described in human osteosarcoma 32. To document P2RX7B expression, the authors used a differential screening approach, based on two antibodies, the first one recognizes both P2RX7A and P2RX7B, whereas the second is specific to P2RX7A only. Thank to this approach, specimens positively labeled with the first antibody and negatively labeled with the second were described to be positive for P2RX7B. Considering that there is no antibody specific to P2RX7B, this technic represents a smart way to document P2RX7B expression. However, each antibody has his own affinity for its specific epitope and from this affinity depends the strength of the signal. Therefore, a subtractive strategy to qualify the expression of P2RX7B is a tricky approach to handle. To overcome this limitation, the authors further transfected Te85 osteosarcoma cell line with either P2RX7A, P2RX7B or both. Doing so, they demonstrated that cells expressing both isoforms were more proliferative and more prone to induce mineralization. More recently, expression of P2RX7B has been described to be upregulated during the osteogenic process 18. Evidence suggesting that P2RX7A and P2RX7B might heteromerized were obtained in the X. *laevis* oocyte expressing system after co-immunoprecipitation of tagged P2RX7A and P2RX7B 31. In addition, transfection of HEK cells with both P2RX7A and P2RX7B suggested that P2RX7B disguises P2RX7A's cytotoxic activity. If true, expression of P2RX7B will give a growth advantage and explain how P2RX7 expression (likely A and B isoforms) could have been linked to tumor growth and invasiveness in several cancer types 36-39. Indeed, considering that P2RX7 is a pro-apoptotic receptor, it is counterintuitive to imagine that tumor cells express a receptor capable of inducing their own death. It has been proposed that tumor cells express a non-conformal receptor (nfP2RX7), which is unable to induce cell death but retains calcium channel activity and therefore could promote tumor growth. Herein, we observed that P2RX7 expressed by tumor cells, but also normal epithelial cells, lacked the macropore function, whereas immune cells retained it. At this time, we still do not know whether the isoform of P2RX7 expressed by tumor cells corresponds to nfP2RX7, since the only way to characterize its expression relies on the use of an anti-nfP2RX7 antibody, which is not commercially available. We explored the hypothesis that the lack of macropore activity in tumor and non-tumor epithelial cells could be the consequence of expression of truncated P2RX7 isoforms, resulting from alternative splicing of P2RX7 mRNA rather than SNPs. Indeed, allele frequency of SNPs located to the boundary of mRNA splicing regulation sites within intronic regions is too low to envisage that these SNPs could explain the significant dysfunction in P2RX7 that we observed in 10 LUAD patients. Our study revealed for the first time that 3 alternative splice variants of *P2RX7* are expressed in LUAD patients, namely *P2RX7B, P2RX7H* and *P2RX7J*. We also observed that all *P2RX7* variants were down-regulated by at least 2.5-fold in tumor tissues versus adjacent non-tumor tissues. Since each *P2RX7* variant was down-regulated to the same extend in non-immune cells, we believe that the lack of P2RX7 function is a consequence of decreased protein expression rather than the formation of hetero trimers that may be stored in intracellular vesicles, as it was proposed for nfP2RX7 40.

Whereas each *P2RX7* variant was equally down-regulated in the epithelial compartment of tumor tissues versus adjacent non-tumor tissues, we observed differential up-regulation of P2RX7B in the tumor immune compartment. Over expression of *P2RX7B* has already been reported in *ex vivo* mitogenic-induced immune cells, in particular in lymph nodes and lymphocytes 31, and the authors proposed that expression of P2RX7B correlated with lymphocyte proliferation. Our results do not support this proposition since we showed that the more *P2RX7B* is expressed the less the tumors were infiltrated by T and B lymphocytes. Besides suggesting a physiological role for the truncated P2RX7B isoform, this result questions how P2RX7B expression can impact on the immune infiltrate composition. Using the bi-molecular fluorescent approach, we confirmed that a chimeric P2RX7AB receptor is formed, and we showed for the first time that a large fraction (63%) was retained intracellularly. To avoid potential bias linked to the presence of the N-terminal venus tag, we co-transfected HEK cells with untagged P2RX7A or P2RX7A+P2RX7B and we selected stable clones expressing comparable protein levels of both isoforms. Doing so, we were able to demonstrate that cells expressing both P2RX7A and P2RX7B isoforms were less prone than cells expressing only P2RX7A to form a macropore, whereas they retained the same ion channel capability. This result does not confirm what was previously published indicating that P2RX7B expression positively modulates P2RX7 function 31 but they confirm that P2RX7B expression could disguise P2RX7's macropore activity 19,33. Whether this alteration in macropore function observed *ex vivo* is due to the coexistence of cells expressing only homo trimeric P2RX7B and only homo trimeric P2RX7A, or to the existence of cells expressing intracellular chimeric P2RX7AB, or both remains to be determined. However, our results showed that tumors of LUAD patients with a high *P2RX7B* expression were less infiltrated with B and T cells and more infiltrated with myeloid cells, suggesting that differential expression of *P2RX7B* critically regulated the quality of tumor immune cell infiltration. Whether differential expression of *P2RX7B* results from tumor conditioning of the lung tissue or specific expression in immune cells before tumor conditioning is still an open question. Nevertheless, considering that expression of *P2RX7B* in LUAD correlated with both an alteration in the P2RX7 function and the lesser infiltrated tumor phenotype (also called “cold” tumor), it is tempting to propose that P2RX7B participates in tumor development and may therefore represent an attractive theranostic tool.

## Supplementary Material

Supplementary figures and tables.

## Figures and Tables

**Table 1:** Main epidemiological data of LUAD patients

Retrospective study (n=29)		
Age (years)	Mean (Range)	67 (41 - 85)
Sex	Female	6 (20.7%)
	Male	23 (79.3%)
Smoking history	Smokers or smoking history	29 (100%)
	Non smoker	0 (0%)
COPD #	Yes	18 (62%)
	No	11 (37.9%)
Stage at surgery	I	15 (51.7%)
	II	10 (34.4%)
	III	2 (6.9%)
	IV	0 (0%)
Adjuvant chemotherapy	Yes	3 (10.3%)
	No	26 (89.7%)
Relapse	Yes	7 (24.1%)
	No	19 (65.6%)
	Unknown	3 (10.3%)
**Prospective study (n=24)**		
Age (years)	Mean (Range)	68 (44 - 80)
Sex	Female	12 (50%)
	Male	12 (50%)
Smoking history	Smokers or smoking history	19 (79.2%)
	Non smoker	5 (20.8%)
COPD	Yes	6 (25%)
	No	17 (70.8%)
	Unknown	1 (4.2%)
Stage at surgery	I	12 (50%)
	II	4 (17%)
	III	8 (33%)
	IV	0 (0%)
Adjuvant chemotherapy	Yes	9 (37.5%)
	No	15 (62.5%)
Relapse	Yes	5 (20.8%)
	No	11 (45.8%)
	Unknown	8 (33.3%)

# COPD: chronic obstructive pulmonary disease.
