# Supplementary material for: P2RX7B is a new theranostic marker for lung adenocarcinoma patients
Source: Theranostics. 2020 Aug 29;10(24):10849–60. doi: 10.7150/thno.48229 (PMC7532666; doi:10.7150/thno.48229)

Supplementary Figure 1: Separation of cell populations from LUAD tissues

A. Graphical representation of the isolation of cell populations. **B.** Quality control after cell separation. Sorted $CD45^+$ and $CD45^-$ cells from non-tumor (N) and tumor (T) areas of LUAD tissues were stained with an anti-CD45 antibody and analyzed by flow cytometry. n=3 independent experiments.

Supplementary Figure 2: Expression of the homo-P2RX7A trimer

hP2RX7 HEK or purified $CD45^-$ cells were permeabilized or not with 1% triton and stained with the conformational anti-P2RX7 antibody to characterize intracellular (intra) and membrane expression (Mb) of P2RX7. This result showed that $CD45^-$ cells from a tumor area of LUAD patients did not retained P2RX7 within the cytoplasm.

Supplementary Figure 3: Characterization of P2RX7 activity

A. Representative dot plots are shown. hP2RX7 HEK cells were stimulated for 5 min with the indicated doses of BzATP (a stable analogue of ATP) and stained with both TO-PRO-3 (APC) and Fluo-4-AM (FITC) to assay for large pore opening and intracellular Ca^{2+} variations, respectively. **B.** Dose response curves showing large pore opening (TO-PRO-3 uptake) and Ca^{2+} channel activation in response to BzATP in live cells. n=3 independent experiments.

Supplementary Figure 4: P2RX7 activity in LUAD patients

Representative dot plot showing a time course of macropore opening in purified $CD45^+$ cells (**A**) or $CD45^-$ cells (**B**) isolated from LUAD tissue. The percentage of TO-PRO-3 positive cells in response to BzATP (250 µM) is shown. To demonstrate that P2RX7 is involved in the TO-PRO-3 uptake, cells were pretreated with a specific P2RX7 antagonist (GSK13700319A) 30 min before adding BzATP for an additional 30 min.

Supplementary Figure 5 : *P2RX7* splice variant expression in human peripheral blood mononuclear cells

A. Primers used to analyze *P2RX7* splice variant expression in human peripheral blood mononuclear cells (PBMC). **B.** Representative image showing qualitative PCR. We were unable to generate primers specific for *P2RX7D* and *E*.

Supplementary Figure 6 : Primers used in quantitative PCR experiments

A. Primers used to analyze *P2RX7* splice variant expression by qPCR. **B.** Expression vectors coding for human *P2RX7A*, *P2RX7B*, *P2RX7H* and *P2RX7J* were used to produce specific amplicons of each *P2RX7* mRNA. The number of molecules was calculated and serial dilutions, to verify the linear amplification of each mRNA after qPCR, were performed.

Supplementary Figure 7: *P2RX7* expression in LUAD

P2RX7 expression obtained from the TCGA database showing down-regulation of P2RX7 in LUAD tissues (T) versus non-tumor tissues (N). *P2RX7* expression was in compared LUAD tissues and adjacent non-tumor tissues. All stage: 57 LUAD tissues and paired non-tumor tissues; Stage I-II: 42 LUAD tissues and paired non-tumor tissues; Stage III-IV: 15 LUAD tissues and paired non-tumor tissues (unpaired Student's *t* test).

Supplementary Figure 8: Characterization of tagged P2RXA and P2RXA AB receptors

A. Hemi-venus expressing cells are not GFP positive. HEK cells were transiently transfected with P2RX7A and P2RX7B tagged with venus 1 or venus 2 sequences. Representative images of P2RX7B-v1, P2RX7B-v2, P2RX7A-v1 and P2RX7A-v2 showing that only cells expressing the trimeric conformation of P2RX7A are stained with the anti-P2RX7. P2RX7 (red), v1-v2 complementation (green), nucleus (blue). **B.** Representative illustration of the quantification of v1-v2 (GFP) and P2RX7A (red) positive cells. The distance between tagged-P2RX7 and P2RX7A was calculated using Image J software.

Supplementary Figure 9: Characterization of P2RX7B isoform in HEK transfected cells

A. HEK cells were transfected with empty vector, P2RX7A or P2RX7B. 50 ug of total protein were analyzed by western blotting using the anti-extracellular loop anti P2RX7 antibody. In cells transfected with P2RX7A we observed a band of 70 kDa. In cells transfected with P2RX7B, we observed two bands, one of 55 kDa which corresponds to P2RX7B and one at lower molecular which likely corresponds to

non-glycosylated protein. Expression of two bands in cells transfected with P2RX7B were previously observed in Adinolfi et al, 2010. **B.** Immunofluorescence analysis of HEK cells expressing either P2RX7A or P2RX7B. Cells were stained with the conformational anti-P2RX7 antibody (left panel). These results showed that P2RX7B is not recognized by the conformational antibody. The right panel illustrates HEK cells transfected with v1+v2-P2RX7B showing GFP fluorescence at the membrane (asterisk). **C**. P2RX7B expressed in HEK cells is activated by BzATP and induced increased Ca^{2+} concentration but not large pore opening. n=4 independent experiments.

Supplementary Figure 1

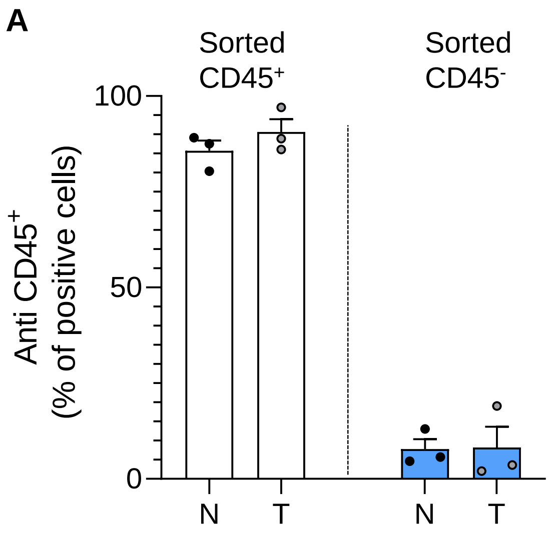

Supplementary Figure 2

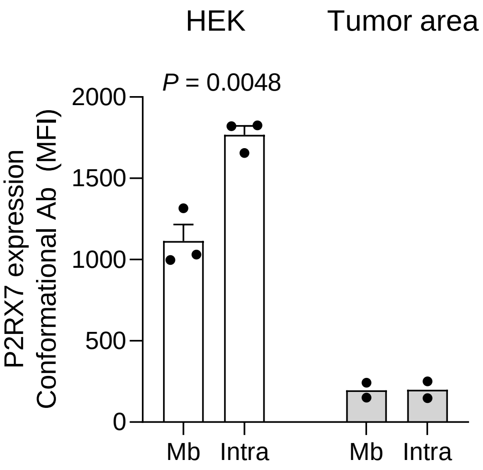

Supplementary Figure 3

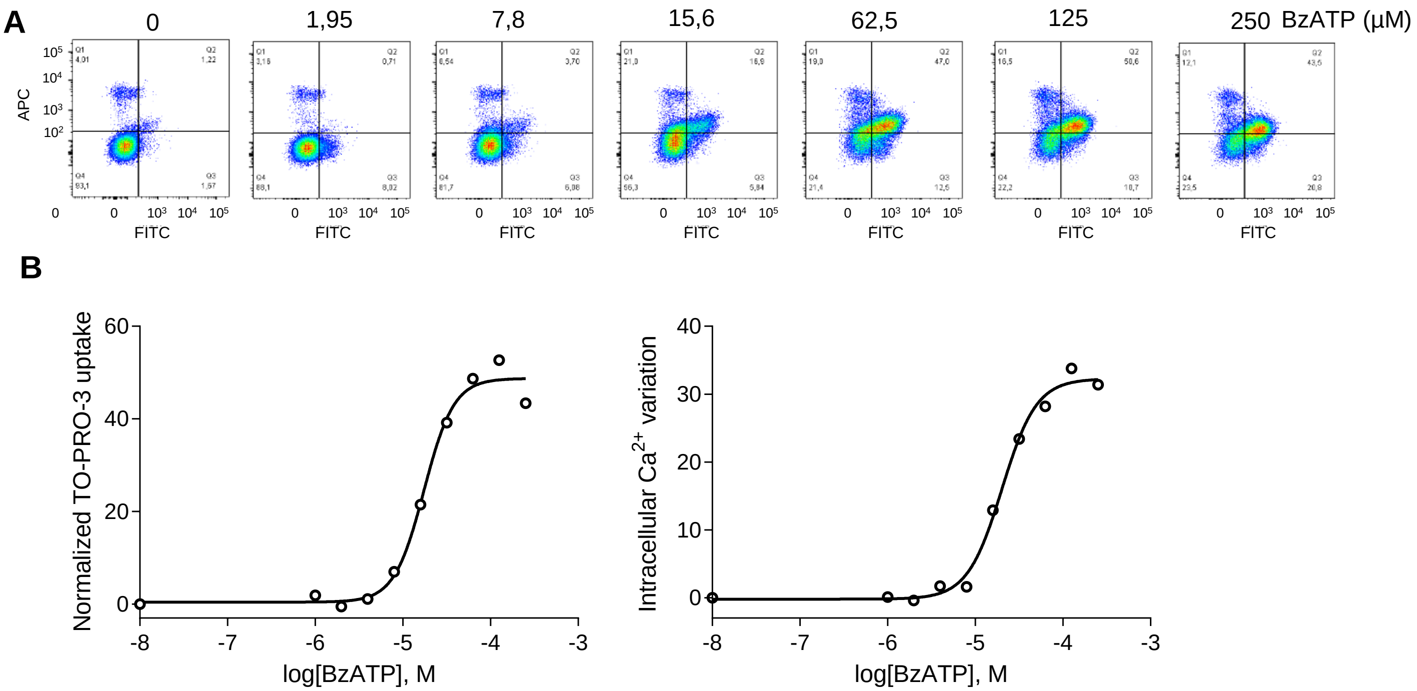

Supplementary Figure 4

A

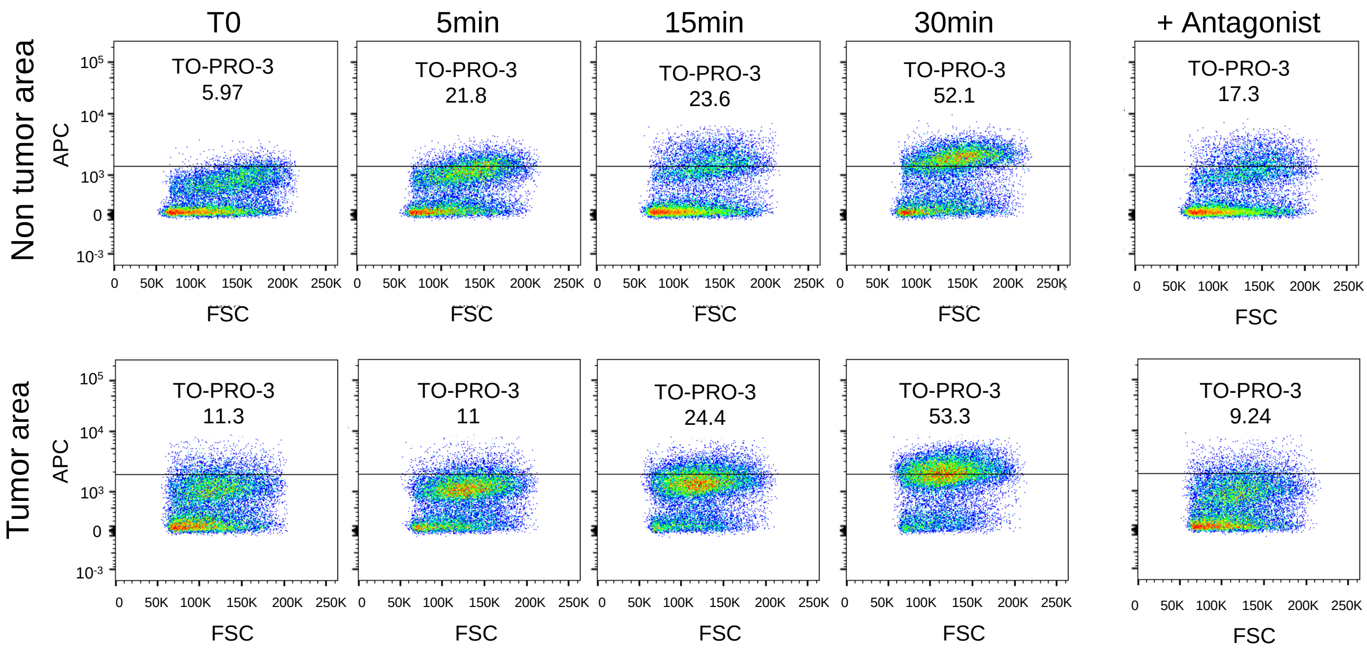

B

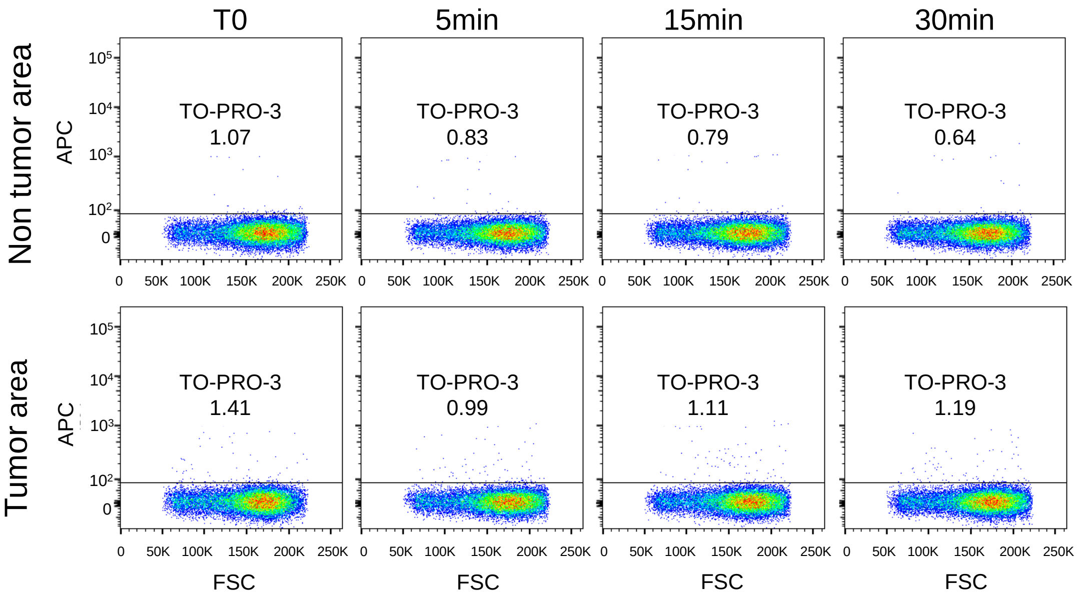

Supplementary Figure 5

A

Specificity	Forward primer	Reverse primer	MW
P2RX7(A, B, H)	**GAACCAGCAGCTACTAGGGAGAAG**	**GAACCAGCAGCTACTAGGGAGAAG**	476
P2RX7B	**CGGCCACAACTACACCACGAG**	**CGGCCACAACTACACCACGAG**	527
P2RX7H	**CAAGGTCAGCCGAGATTCAG**	**CAAGGTCAGCCGAGATTCAG**	850
P2RX7J	**TTTCAGATGTGGCAATTCAGATA**	**TTTCAGATGTGGCAATTCAGATA**	150

B

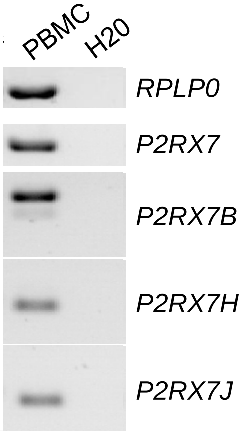

Supplementary Figure 6

A

Specificity	Forward primer	Reverse primer	MW
P2RX7 (A, B, H)	ATACAGTTTCCGTCGCCTTG	AACGGATCCCGAAGACTTTT	133
P2RX7B	GACATTATCCAGCTGGTTG	GACAAGCGCTGCGTTAGTCAC	125
P2RX7H	CAAGGTCAGCCGAGATTCAG	CAAGGTCAGCCGAGATTCAG	111
P2RX7J	TTTCAGATGTGGCAATTCAGATA	TTTCAGATGTGGCAATTCAGATA	150

B

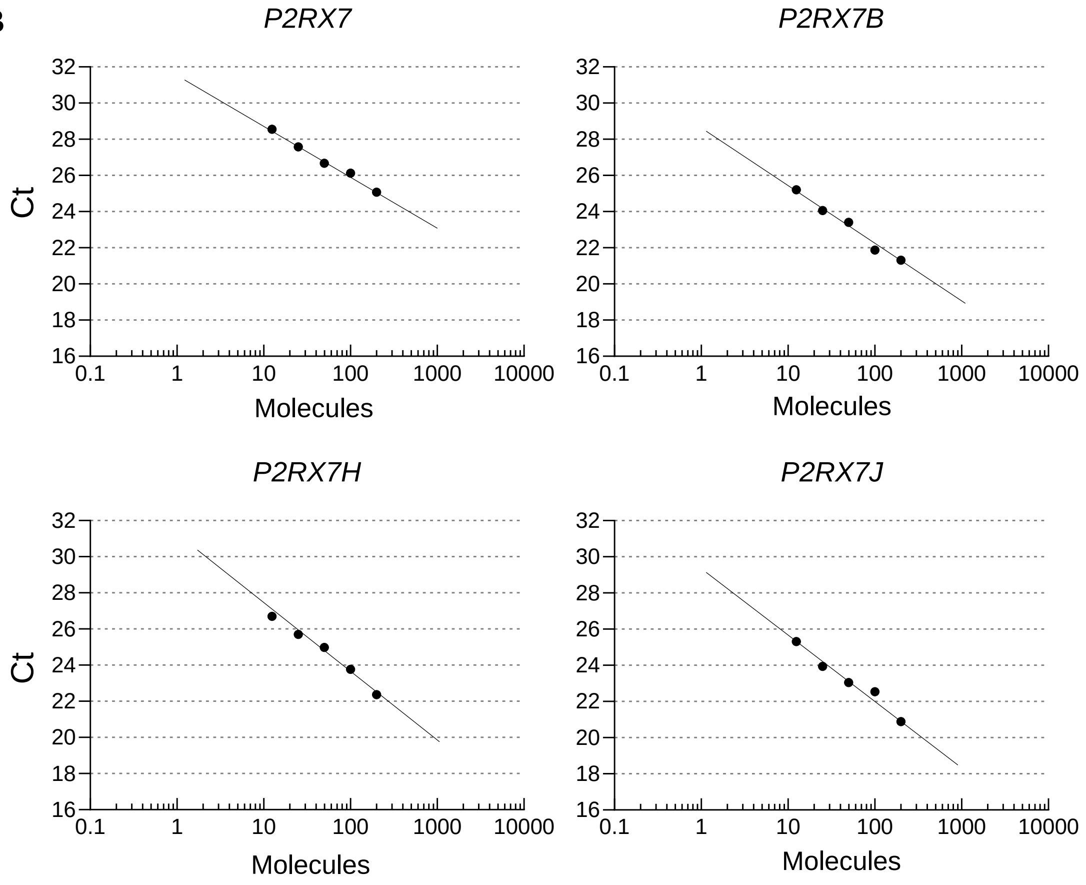

Supplementary Figure 7

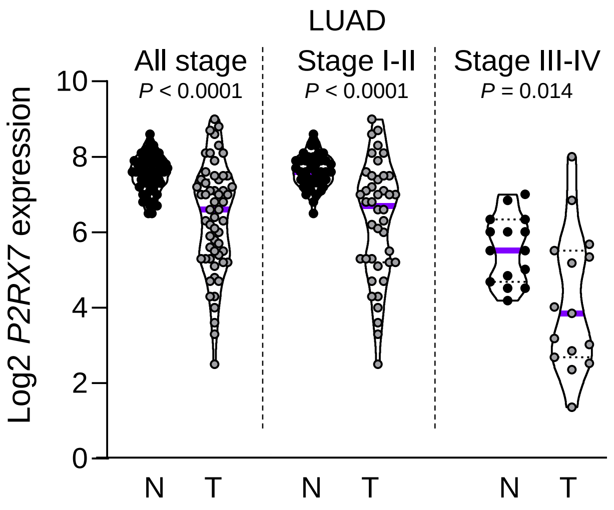

Supplementary Figure 8

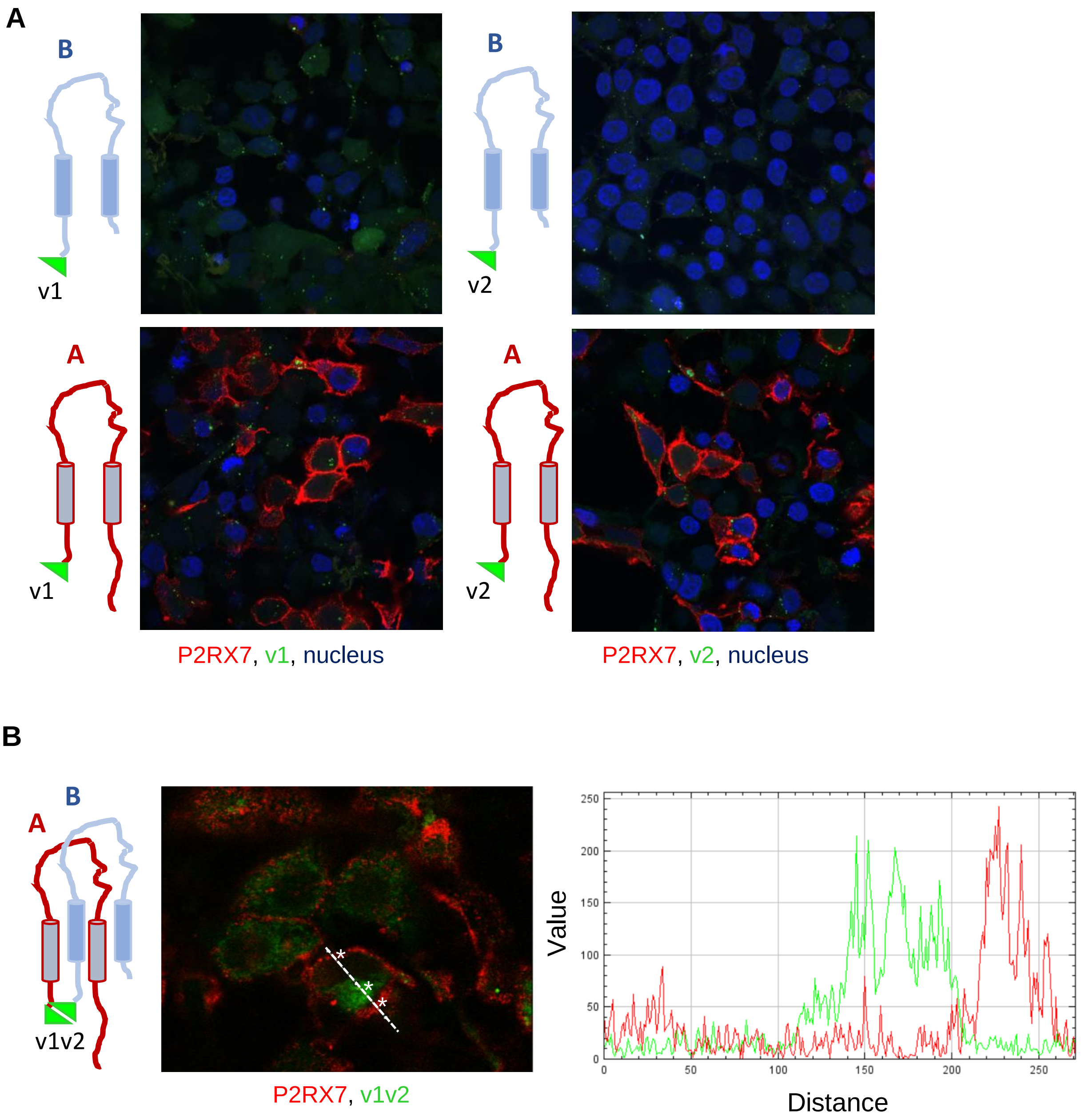

Supplementary Figure 9

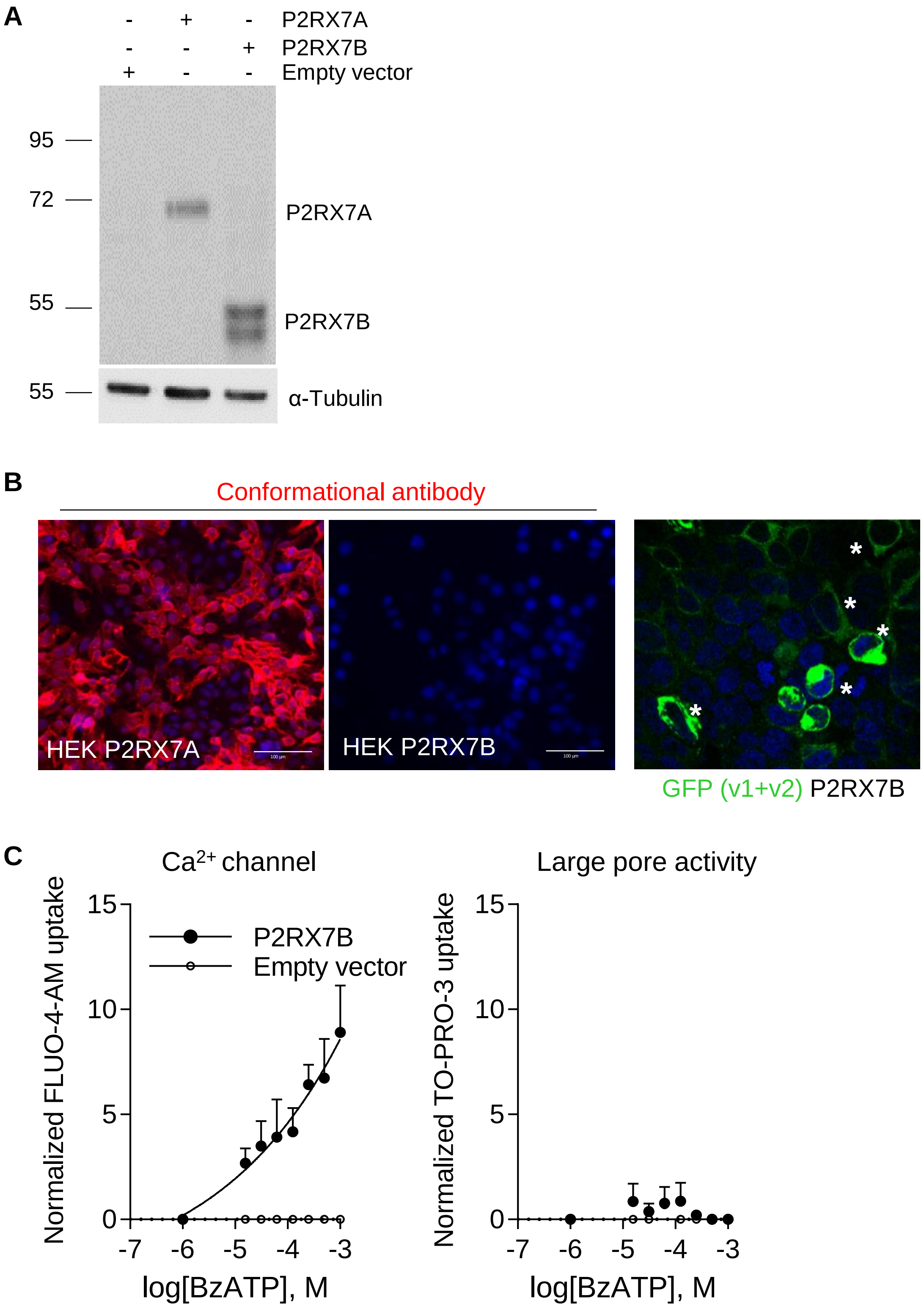

Supplement: Supplementary file 1 — Supplementary figures and tables. [file thnov10p10849s1.pdf]
